# Parkinson’s Disease Rigidity: Relation to Brain Connectivity and Motor Performance

**DOI:** 10.3389/fneur.2013.00067

**Published:** 2013-06-05

**Authors:** Nazanin Baradaran, Sun Nee Tan, Aiping Liu, Ahmad Ashoori, Samantha J. Palmer, Z. Jane Wang, Meeko M.K. Oishi, Martin J. McKeown

**Affiliations:** ^1^Pacific Parkinson’s Research Centre, University of British Columbia, Vancouver, BC, Canada; ^2^Department of Electrical and Computer Engineering, University of British Columbia, Vancouver, BC, Canada; ^3^Department of Electrical and Computer Engineering, University of New Mexico, Albuquerque, NM, USA; ^4^Department of Medicine (Neurology), University of British Columbia, Vancouver, BC, Canada

**Keywords:** Parkinson’s disease, rigidity, fMRI, damping ratio, linear dynamical system, LASSO regression

## Abstract

**Objective:** (1) To determine the brain connectivity pattern associated with clinical rigidity scores in Parkinson’s disease (PD) and (2) to determine the relation between clinically assessed rigidity and quantitative metrics of motor performance.

**Background:** Rigidity, the resistance to passive movement, is exacerbated in PD by asking the subject to move the contralateral limb, implying that rigidity involves a distributed brain network. Rigidity mainly affects subjects when they attempt to move; yet the relation between clinical rigidity scores and quantitative aspects of motor performance are unknown.

**Methods:** Ten clinically diagnosed PD patients (off-medication) and 10 controls were recruited to perform an fMRI squeeze-bulb tracking task that included both visually guided and internally guided features. The direct functional connectivity between anatomically defined regions of interest was assessed with Dynamic Bayesian Networks (DBNs). Tracking performance was assessed by fitting Linear Dynamical System (LDS) models to the motor performance, and was compared to the clinical rigidity scores. A cross-validated Least Absolute Shrinkage and Selection Operator (LASSO) regression method was used to determine the brain connectivity network that best predicted clinical rigidity scores.

**Results:** The damping ratio of the LDS models significantly correlated with clinical rigidity scores (*p* = 0.014). An fMRI connectivity network in subcortical and primary and premotor cortical regions accurately predicted clinical rigidity scores (*p* < 10^−5^).

**Conclusion:** A widely distributed cortical/subcortical network is associated with rigidity observed in PD patients, which reinforces the importance of altered functional connectivity in the pathophysiology of PD. PD subjects with higher rigidity scores tend to have less overshoot in their tracking performance, and damping ratio may represent a robust, quantitative marker of the motoric effects of increasing rigidity.

## Introduction

Rigidity is defined by increased resistance during passive mobilization of an extremity, independent of direction and velocity of movement (Delwaide, 2001), and is one of the cardinal diagnostic features of Parkinson’s disease (PD), along with tremor, bradykinesia, and postural instability (Tolosa et al., 2006; Shapiro et al., 2007). Since rigidity can be a manifestation of various pathologies involving the basal ganglia and can be altered during states of drowsiness or relaxation (Webster, 1960; Fung and Thompson, 2007), it is usually not considered pathognomonic of PD.

The underlying mechanism of rigidity in PD is poorly understood, and no direct relationship exists between dopamine deficiency and rigidity, making it difficult to explain through the classic model of basal ganglia pathophysiology (Rodriguez-Oroz et al., 2009). The classic description of basal ganglia activity in PD predicts that increased neuronal activity in the subthalamic nucleus (STN) and internal globus pallidus (GPi), and its resultant inhibition of thalamocortical projections, should result in *decreased* muscle activation and reduced response to stretching when, in fact, the opposite is observed (Bezard and Przedborski, 2011).

Contributions from the spinal cord, brain stem including higher cortical circuits have all been proposed as being important in the pathophysiology of rigidity (Hong et al., 2007), and several mechanisms, likely not mutually exclusive, may be responsible (Delwaide, 2001). One potential mechanism may be increases in excitability in long loop reflex pathways. Rapid stretching of a contracting muscle results in responses at different latencies. The most rapid response corresponds to the well-known monosynaptic involuntary stretch reflex easily assessed by tapping a tendon with a reflex hammer. A longer latency response corresponds to transcortical involvement. It is postulated that if this transcortical loop is hyperactive, then enhanced response to stretching may appear clinically as rigidity. A second postulate suggests that inappropriate commands from one or several descending spinal pathways caused malfunctions of the short reflex pathways at the spinal level (Delwaide, 2001). However, clinical observations may suggest an alternate explanation. It is frequently observed that Froment’s maneuver (voluntary movement of the contralateral limb) can accentuate or even unmask latent rigidity. This implies that a systems-level, distributed brain network may contribute significantly to the mechanism of rigidity in PD. Therefore here we utilize fMRI imaging techniques to determine distributed brain connectivity patterns that predict clinical rigidity scores.

While PD patients may complain of stiffness, or even present with functional limitation (e.g., “frozen” shoulder), in general, rigidity is a sign detected by the clinician rather than a symptom described by patient. Yet, despite its potential functional importance, the implications of progressive rigidity in PD on quantitative motor performance are not currently known. Here we utilize Linear Dynamical System (LDS) models of tracking behavior collected concomitantly during the fMRI scanner session to assess motor performance, as we have previously shown this to be a more sensitive measure of motor performance than the normally used overall tracking error (Oishi et al., 2011). When PD subjects are asked to track a target, they tend to undershoot the actual target (Van Gemmert et al., 2003). This undershooting is rigorously defined as “damping ratio” in control systems. Specifically the damping ratio describes the behavior of a system tracking a desired target. Highly underdamped systems tend to oscillate around the desired trajectory, where highly overdamped systems tend to be sluggish and slow, and fail to sufficiently track rapidly changing targets (Ljung and Ljung, 1987). We thus hypothesize that the damping ratio parameter of LDS models fitted to each subject’s motor performance would closely correlate with overall clinical rigidity scores.

## Materials and Methods

### Subjects

Written, informed consent was obtained from all subjects in accordance with the Declaration of Helsinki, and the study was approved by the University of British Columbia Research Ethics Board. Ten subjects with clinically diagnosed PD (off-medication) and 10 healthy age-matched control subjects were recruited from the Pacific Parkinson’s Research Centre (PPRC)/Movement Disorders Clinic. In the PD group, all subjects (four men, six women, eight right-handed, and two left-handed) were PD patients diagnosed with mild to moderate PD (Hoehn and Yahr stage 2–3) (Hoehn and Yahr, 1967). Their mean symptom duration and mean age were 5.8 ± 3 years and 66 ± 8 years, respectively. PD subjects stopped their l-dopa medications overnight for a minimum of 12 h before the study. Those who were also taking dopamine agonists were withheld from medications for a minimum of 18 h. The mean Unified Parkinson’s Disease Rating Scale (UPDRS) motor score off-medication was 26 ± 8 (Table 1).

**Table 1:** **Demographic characteristics of PD patients and normal healthy controls**.

Demographic characteristics	PD	Nl
Gender
Male	4	3
Female	6	7
Handedness
RH	8	9
LH	2	1
Hoehn and Yahr stage	2–3	N/A
Mean symptom duration	5.8 ± 3 years	N/A
Mean age	66 ± 8 years	57.4 ± 14 years
Average daily dose of l-dopa	685 ± 231 mg	N/A
Mean UPDRS	26 ± 8	N/A

Additionally, we recruited 10 healthy, age-matched individuals (three men, seven women, nine right-handed, one left-handed) without active neurological disorders as control subjects with mean age of 57.4 ± 14 years. Our exclusion criteria were: (1) subjects presenting with atypical Parkinsonism, (2) presence of other neurological or psychiatric conditions, (3) use of antidepressants, hypnotics, or dopamine blocking agents. All PD subjects were taking l-dopa medication with an average daily dose of 685 ± 231 mg, and additionally some subjects took other anti-Parkinson’s medications, including ropinirole, bromocriptine, and domperidone. For the 3/20 subjects who were left-hand dominant, we still asked subjects to perform the task with their right hand to ensure that lateralized activity in motor regions (e.g., cerebellar hemisphere, primary motor cortex) was relatively consistent. While complex hand sequence movements tend to be lateralized to the left hemisphere (Lotze et al., 2000), independent of the hand moving, lateralization was more strongly dependent upon the actual hand used since our task was simple and over-learned.

### Experimental design

**Figure 1:**
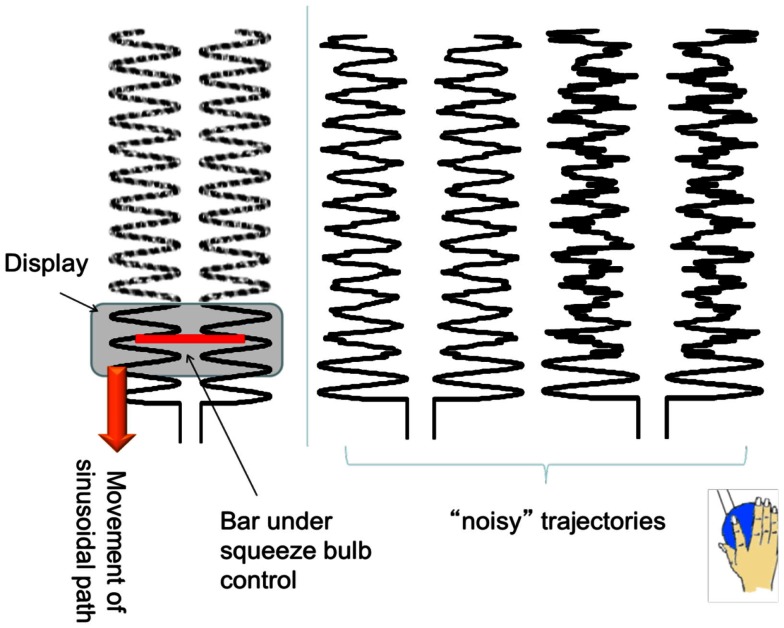
**An illustration of the experimental task**. The sinusoidal path scrolls down vertically, with different frequencies of noise trajectories. Subjects had to control the width of the (red) bar to maintain the ends of the bar within the sinusoidal pathway.

To ensure that the results we found were relatively robust to the specific task performed, we purposely chose a task that included both externally guided (EG, e.g., in response to a visual stimulus) and internally guided (IG, e.g., recalled from memory) aspects. The basal ganglia are more active when a subject must perform an action that is selected from many potential candidates of action (Mushiake and Strick, 1995; Jueptner and Weiller, 1998; van Donkelaar et al., 1999, 2000). The cerebellum, traditionally associated with pure motor control, is now considered to be essential for the development of “forward models,” such as predicting the sensory consequences of motor actions (Blakemore et al., 2001; Miall and Jenkinson, 2005). Cerebellar activity is normally associated with EG movements where sensorimotor integration is important (Jueptner et al., 1996; van Donkelaar et al., 1999, 2000). Therefore the task consisted of a squeezing a bulb in a sinusoidal pattern that was guided by visual cues corrupted with varying amounts of noise. Specifically, subjects were instructed to squeeze a rubber bulb with their right hand to control the width of a bar, which did not translate horizontally or vertically. Subjects were asked to keep the ends of the black bar within a 0.5 Hz vertically scrolling pathway by squeezing the bulb which required a force between 5 and 15% maximum voluntary contraction (MVC) (see Figure 1 for illustration of the task). They were asked to maintain a smooth sinusoidal force pattern at 0.5 Hz even when the scrolling pathway was partially degraded by varying amount of noise levels (0, 25, and 50%). Since we were interested in examining altered connectivity patterns, subjects performed 90 s runs where the noise level was kept constant. Each subject performed three 90 s runs at each of the three noise levels. The rubber squeeze-bulb was a custom-built, in-house designed system connected via water-filled, low-compliance tubing to a precision pressure transducer (Honeywell, Inc., Plymouth, MN, USA; model PPT0100AWN2VA) outside the scanner room. Each subject’s MVC was assessed at the beginning of a 30-min training session by asking them to squeeze the bulb with their maximum force for 15 s while the pressure was measured. The median pressure over the 15 s was used as the MVC. All visual stimuli were coded with Matlab (Natick, MA, USA) and the Psychtoolbox (Brainard, 1997).

### Behavioral data analysis

Behavioral force data from the squeeze-bulb were sampled at ∼50 Hz. We first computed the root mean square (RMS) error between the actual and desired (pure sinusoidal) squeezing profiles. For a pursuit tracking task with input trajectory *u* and output trajectory *y*, the RMS error is calculated as:

(1) $$E_{RMS}=\sqrt{\frac{1}{N}\sum_{i=1}^{N}{\left(y[i]-u[i]\right)}^{2}},$$

where *u*[*i*] is the desired position at time index *i*, and *y*[*i*] is the actual tracking done by the individual at time index *i*, and *N* is the number of time points.

We used System Identification techniques to assess tracking performance in PD. A standard discrete second-order linear dynamical system model is defined as, **x***_t_* = **Ax***_t-1_* + **Bu***_t-1_*; y*_t_* = **Cx***_t_* + **D**u*_t_*; where u*_t_* represents the desired sinusoidal trajectory and y*_t_* represents the actual bar width at time *t*. From these two sets of values, the constant matrices **A**, **B**, **C**, and **D** can be extracted. It is important to note that these matrices completely characterize all possible system responses, that is, once tracking performance is successfully modeled, then the output *y_t_* can be predicted for any given input *u_t_*, not just those that were chosen experimentally. Previous work, including our own, has suggested that second-order models can successfully model normal and PD subjects during a tracking task (Oishi et al., 2011).

Since the system response, *y_t_* depends on the eigenvalues of **A**, the eigenvalues can capture the essential features of each model. However, in order to make the characterizations of the models more intuitive, it is customary to transform the eigenvalues into two parameters: damping ratio (ζ) and natural frequency (ω_n_), such that $\lambda_{1,2}=-\zeta \omega_{n}\pm \left(\omega_{n}\right)\sqrt{\zeta^{2}-1}$. A higher damping ratio is usually associated with a better performance, i.e., less oscillation and overshoot around the desired trajectory, with lower damping ratio associated with less damping (and more overshoot) in the error response. The natural frequency does not necessarily reflect that speed at which the subject was moving, rather it reflects the responsiveness of the system: a higher natural frequency is associated with faster response; while lower natural frequency is associated with slower response. Since we were interested in determining if rigidity had a linear correlation with one or more movement parameters, we also computed other parameters derived non-linearly from the eigenvalues, including rise time, peak time, and settling time.

### Clinical rigidity scores

The same trained operator evaluated all PD patients in the off-medication state, to obtain the clinical rigidity score using part three of the UPDRS. A global rigidity score was estimated by simply summing the individual limb and truncal rigidity scores.

### Data acquisition

The MRI data were collected from a Philips Achieva 3.0 T scanner (Philips, Best, Netherlands) equipped with a headcoil. A whole brain three-dimensional T1-weighted image consisting of 170 axial slices with high resolution were also acquired to facilitate the anatomical localization for each individual. Blood oxygenation level-dependent (BOLD) contrast echo-planar (EPI) T2*-weighted images were taken with the following specifications: repetition time 1985 ms, echo time 37 ms, flip angle 90 °, field of view (FOV) 240.00 mm, matrix size 128 × 128, with pixel size 1.9 mm × 1.9 mm. The duration of each functional run was 4 min during which we obtained 36 axial slices with 3 mm thickness and 1 mm gap thickness. The FOV was set to include the cerebellum ventrally and also include the dorsal surface of the brain.

### fMRI data pre-processing and analysis

Slice time correction, isotropic reslicing of voxels, and initial motion correction was performed with SPM99. We then used custom-built motion-correction software that is particularly accurate for the larger head motion seen in older and PD subjects (Liao et al., 2005, 2006). Low frequency drifts were removed with a discrete cosine transformation, with cutoff period of 128 s. We did not spatially normalize each subject’s data to a common space, as we have demonstrated that this will incur excessive error (Nieto-Castanon et al., 2003; Chen et al., 2009; Ng et al., 2009). Fifty two regions of interest (ROI) were extracted using a combined FreeSurfer (Harvard, MA, USA; http://surfer.nmr.mgh.harvard.edu/) and Large Deformation Diffeomorphic Metric Mapping (LDDMM) method (Khan et al., 2008).

### Connectivity analysis: PCfdr

The connectivity network between 52 FreeSurfer-derived ROIs was computed with the PCfdr (Peter Spirtes and Clark Glymour, false discovery rate) algorithm (Li and Wang, 2009). We selected these 52 ROIs based on motor regions and the ROIs involved in the Default Mode Network (DMN), which has been shown to be altered in PD (van Eimeren et al., 2009; Palmer et al., 2010). The PCfdr method is designed to overcome the typical problem for fMRI experiments, which is a large number of ROIs but relatively few time points. After taking the average time course of all voxels within each ROI (after linear detrending) to get an ROI timecourse, the PCfdr method determines the conditional (in) dependence of each pair of ROIs dependent on all other ROIs to determine if two ROIs are connected. We set the FDR threshold at 5% in this study. In order to aid comparisons, we pooled the PD and control groups together and computed the significant connections amongst ROIs. The subject specific connection strengths were then determined using standard dynamic Bayesian network (DBN) methodology (Li et al., 2008).

### Correspondence between connectivity and clinical rigidity scores

We used multivariate linear regression to determine whether or not clinical rigidity scores could be predicted from the connectivity patterns in PD subjects (“lasso” command in Matlab). Specifically, we modeled the rigidity scores as:

(2) Y=X⋅β+ε

where *Y* was a vector of rigidity scores of dimensions 10 (i.e., number of subjects) by 1, *X* was 10 by *n* (where *n* is the number of significant connections between ROIs determine by the PCfdr/DBN method) and ε is a 10 by 1 vector of residuals. Since, in this case, the number of potential regressors (*n*) exceeds the number of examples (Van Gemmert et al., 2003), we utilized (Least Absolute Shrinkage and Selection Operator) LASSO regression (*lasso* command in Matlab) (Tibshirani, 1996). Unlike other methods such as ridge regression or ordinary least squares, LASSO regression puts a sparsity constraint on β so that most values are zero and attempts to find the most informative connections to predict clinical scores (Tibshirani, 1996). The number of regressors selected by the LASSO operator was to give the least predictive error based on a 10-fold cross-validation. Once the regressors were selected, we used robust regression (*robustfit* command in Matlab) to estimate the significance of the individual regressors.

## Results

### Behavioral data

All PD individuals and healthy age-matched controls successfully conducted the visually guided tracking task at the required frequencies. In PD patients, neither rigidity nor tremor caused any prominent difficulty with task performance. The RMS error did not differ significantly between PD and normal groups [ANOVA (*F*(2, 166) = 1.56, *P* > 0.05)], suggesting that PD subjects were able to robustly perform the task.

### Correlation between clinical rigidity scores and selected motor network

The PCfdr method detected 227 significant connections between ROIs, and thus the X matrix in Eq. 2 was 10 × 227. The 227 significant connections represents ∼8.6% of all possible 52 × 51 = 2,652 directional connections.

**Figure 2:**
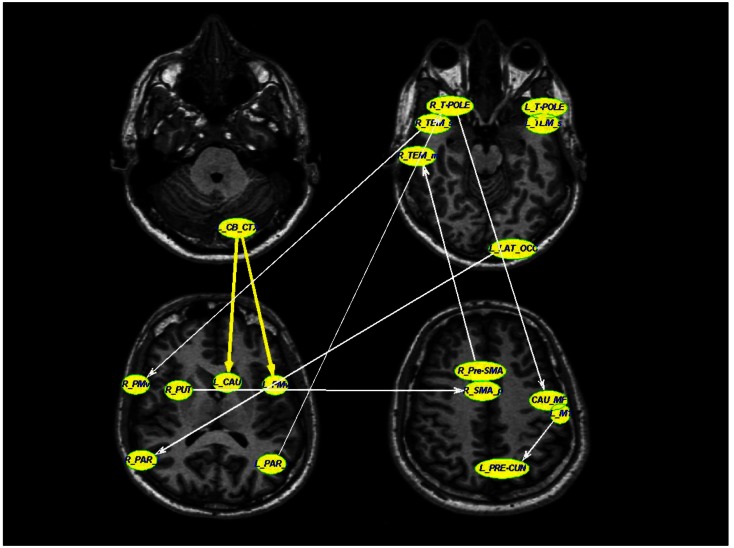
**A schematic diagram depicting the connections that were associated with rigidity in PD**. Thick yellow arrows represent positive correlation between connection strength and rigidity whereas thin white arrows represent negative relationship between connection strength and rigidity (see Table 2 for details of statistical values). Connections with significant positive correlations: 1. From left cerebellar cortex (L_CB_CTX) to left ventral premotor area (L_PMv) (*p* = 0.0002), 2. Left temporal pole region (L_T-POLE) to left superior temporal (L_TEM_s) (*p* = 0.0119). Connections with significant negative correlations: 1. Right superior temporal (R_TEM_s) to right ventral premotor area (R_PMv) (*p* = 0.03), 2. Right putamen (R_PUT) to right supplementary motor area (R_SMA) (*p* = 0.002), 3. Right temporal pole region (R_T-POLE) to left medial frontal caudate (CAU_MF) (*p* < 10^−5^), 4. Left precentral motor area (L_M1) to left pre-cuneus (L_PRE-CUN) (*p* = 0.02), 5. Left lateral occipital (L_LAT_OCC) to right inferior parietal (R_PAR_i) (*p* = 0.02), 6. Right pre-supplementary motor area (R_Pre-SMA) to right middle temporal (R_TEM_m), and 7. Left inferior parietal (L_PAR_i) to right temporal pole region (R_T-POLE) (*p* = 0.03).

The LASSO regression operator selected nine of 227 significant connections between brain regions that significantly predicted rigidity (*p* < 10^−5^). These regions include primary motor area (M1), ventral premotor area, supplementary motor area, basal ganglia, areas in the temporal, parietal, and occipital lobes as well as the cerebellum. The positive and negative correlation between connectivity measures and clinical rigidity scores are demonstrated in Figure 2 and summarized in Table 2.

**Table 2:** **The individual directional connections within this selected network found to significantly correlate with clinical rigidity scores**.

From	To	Sign	*p* Value
*Left cerebellar cortex	Left ventral premotor area	+	0.000176
Right superior temporal	Right ventral premotor area	−	0.002839
Right putamen	Right supplementary motor area	−	0.001756
Right temporal pole region	Left caudal medial frontal gyrus	−	0.000000
*Left primary motor cortex	Left pre-cuneus	−	0.020865
Left temporal pole region	Left superior temporal	+	0.011974
Left lateral occipital	Right inferior parietal	−	0.024952
Right pre-supplementary motor area	Right middle temporal	−	0.022164
Left inferior parietal	Right temporal pole region	−	0.026495

*These two connections had significant stronger connectivity in normal controls compared to PD subjects with significant values of p = 0.007 (left cerebellar cortex → left ventral premotor area) and p = 0.025 [left primary motor cortex (M1) → left pre-cuneus].

Two of the connections within this selected network correlated positively with clinical rigidity scores, i.e., the strength of these connections increased with clinical rigidity scores: 1. Left cerebellar cortex (L_CB_CTX) to left ventral premotor area (L_PMv) (*p* = 0.0002), 2. Left temporal pole region (L_T-POLE) to left superior temporal (L_TEM_s) (*p* = 0.0119). The remaining seven connections within this network were found to correlate negatively with clinical rigidity scores: 1. Right superior temporal (R_TEM_s) to right ventral premotor area (R_PMv) (*p* = 0.03), 2. Right putamen (R_PUT) to right supplementary motor area (R_SMA) (*p* = 0.002), 3. Right temporal pole region (R_T-POLE) to left caudal medial frontal gyrus (L_CAU_MF) (*p* < 10^−5^), 4. Left primary motor area (L_M1) to left pre-cuneus (L_PRE-CUN) (*p* = 0.02), 5. Left lateral occipital (L_LAT_OCC) to right inferior parietal (R_PAR_i) (*p* = 0.02), 6. Right pre-supplementary motor area (R_Pre-SMA) to right middle temporal (R_TEM_m), and 7. Left inferior parietal (L_PAR_i) to right temporal pole region (R_T-POLE) (*p* = 0.03). This implies that the strengths of these seven connections decreased with increasing clinical rigidity scores.

For comparison, we examined the strength of the connections in the rigidity network in PD to the same connections in normal controls. Two of these connections, namely the Left cerebellar cortex (L_CB_CTX) to left ventral premotor area (L_PMv), and also Left primary motor area (L_M1) to left pre-cuneus (L_PRE-CUN) had significantly stronger connections in normal controls compared to PD subjects off-medication (*p* = 0.0076, and 0.025 respectively).

### Correlation of clinical rigidity scores and damping ratio

**Figure 3:**
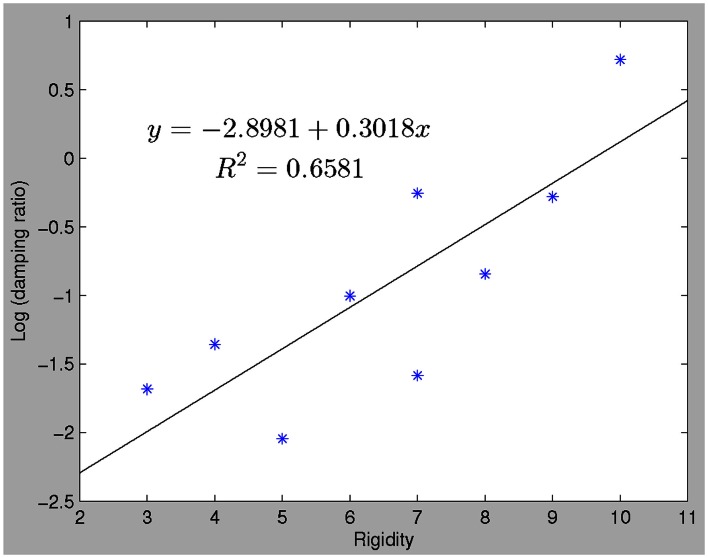
**There is a linear relationship between clinical rigidity scores and damping ratio (robustfit, *p* = 0.0144)**. This relationship could be utilized to predict rigidity scores, which significantly correlate with actual recorded rigidity scores.

The damping ratios of PD subjects had a linear relationship with clinical rigidity scores (*p* = 0. 014) (see Figure 3). No other model parameters significantly correlated with rigidity, including natural frequency and peak time.

## Discussion

We found that clinical rigidity scores are associated with widespread, altered connectivity in subcortical and cortical regions. Several studies also demonstrate altered cortical mechanisms in PD. Transcranial Magnetic Stimulation (TMS) studies suggest increased primary motor cortex excitability in PD (Cantello et al., 1996; Lefaucheur, 2005) at rest. Similarly, MPTP (1-methyl-4-phenyl-1,2,3,6-tetrahydropyridine)-treated monkeys have more vigorous and less specific neuronal responses in the primary motor cortex to passive limb movements. On the other hand, stimulation of the premotor cortex using repetitive TMS increases motor cortex excitability in healthy subjects and PD patients on medication, but fails to do so in patients with PD subjects off dopaminergic medication (Mir et al., 2005). This suggests dopaminergic-dependent defective premotor-motor connectivity in PD that would normally increase motor cortical excitability (Mir et al., 2005). Also, during contraction, there is reduced facilitation of motor response, implying alterations in cortical modulation (e.g., Lefaucheur, 2005). These findings, as well as our own results, further support the notion that dysfunction in higher cortical areas, in addition to subcortical regions, are important for rigidity in PD.

We found a connection from the right putamen to the right SMA that was negatively correlated with clinical rigidity scores (*p* = 0.002). This altered connectivity in the supplementary motor area (SMA) could be considered within the context of the long loop reflex pathway (Berardelli et al., 1983; Delwaide et al., 1986; Delwaide, 2001). The long loop reflex pathway starts from primary endings of the neuromuscular spindles (Ia fibers) carrying action potentials to the spinal cord that travel up the posterior column of the spinal cord to ultimately reach the sensorimotor cortex via the thalamus (Delwaide, 2001). The sensorimotor cortex then sends information back to spinal motor neurons via the corticospinal tract (Delwaide, 2001). Normally, the SMA inhibits the motor cortex. In PD, it has been speculated that the sensorimotor cortex is either facilitated or disinhibited (i.e., in a hyperexcitable state) resulting in increased excitability of the motor cortex (Delwaide et al., 1986; Delwaide, 2001). The long reflex loop may also contain connections from the motor cortex to the basal ganglia, returning to the SMA that then inhibits the motor cortex. In PD, the loop becomes less active, resulting in a hyperexcitable sensorimotor cortex (Delwaide, 2001). Therefore with disease progression, this inhibitory loop becomes less active, resulting in a hyperexcitable motor cortex that manifests as higher rigidity scores in PD patients.

In addition to the SMA → putamen connection observed, which could be part of the long loop reflex, we found connectivity between multiple brain regions that predicted clinical rigidity scores. These included the primary motor area (M1), ventral premotor area, supplementary motor area, basal ganglia, areas in the temporal, parietal, and occipital lobes as well as the cerebellum. The directional connection from the left cerebellar cortex was positively correlated with clinical rigidity scores, consistent with previous observations indicating maladaptive interactions between cerebellar and basal ganglia circuits associated with the increase tone of dystonia (Neychev et al., 2008). Our results revealed a significant negative correlation (*p* = 0.02) between the clinical rigidity score and the connection from the left M1 to the left pre-cuneus. This finding is consistent with previous well-established studies indicating a dysfunctional DMN in PD patients (van Eimeren et al., 2009) and the pivotal role of pre-cuneus in DMN (Fransson and Marrelec, 2008). It has been shown that DMN has a decreased tendency to disengage in PD individuals during an active task (van Eimeren et al., 2009). The negative correlation of the primary motor area to the DMN via the pre-cuneus in our study suggests a disconnection between these cortical regions in PD. The higher the rigidity scores in our patient population, the weaker the connection between M1 and pre-cuneus, suggesting an abnormal communication between the motor system and the DMN in PD population.

A number of connections that were associated with rigidity in our study, while relevant to PD pathophysiology, may reflect a correlative as opposed to a causative relation with rigidity. For example, the temporal pole to superior temporal sulcus connection found in our study may be related to the extensive literature on facial emotion recognition impairments in PD patients (Sprengelmeyer et al., 2003; Suzuki et al., 2006; Lawrence et al., 2007; Clark et al., 2008). The temporal pole has also been suggested to be associated with processing of complex perceptual and emotional stimuli (Olson et al., 2007). A voxel-based morphometry study found significant white matter loss in the superior temporal pole region in PD patients with depression only (Feldmann et al., 2008; Kostic and Filippi, 2011). Thus, depression in PD may be a form of “disconnection” syndrome between neocortical-ventral limbic structures (Kostic and Filippi, 2011). This significant connection was unexpected, and since depression score was not taken into account in this particular study, our finding suggests future studies ought to consider including depression assessment tools.

When we looked at the rigidity network, we found two connections that were of significantly reduced values in PD subjects compared to controls: the left cerebellar cortex (L_CB_CTX) to left ventral premotor area (L_PMv), and also left primary motor area (L_M1) to left pre-cuneus (L_PRE-CUN). What is of particular interest is that the former connection was positively correlated with rigidity, while the latter was negatively correlated. In effect, the cerebellar → premotor connection approached normal values with worsening rigidity. We interpret this as a compensatory mechanism. This is in contrast to the premotor → pre-cuneus connection that became more abnormal with progressive rigidity, and thus was more typical of a direct disease related change.

We used functional connectivity to explore the pathophysiology of rigidity, but is important to appreciate that functional connectivity can be observed between regions where no structural connectivity exists. At the temporal resolution of the fMRI there may be polysynaptic connections between two regions that may make them appear co-activating instantaneously. For example, the functional connection between the right temporal pole region to the left caudal medial frontal gyrus in this study has no known structural connections. Previous studies have suggested that functional connections between two regions that are not anatomically connected may be indicative of mutual influence by a third region (Damoiseaux and Greicius, 2009). We note that the connectivity methods we employed are specifically designed to deal with this possibility.

We observed a robust correlation between damping ratios of the LDS models derived from the behavioral data and clinical rigidity scores (Figure 3), suggesting that damping ratio may be a quantitative surrogate of rigidity. Clinical assessment of rigidity in PD and parkinsonian patients is largely qualitative, whereby a clinician will manipulate the patient’s limb and rate the resistance according to an ordinal rating scale such as that done in the UPDRS. Several studies have examined the inter-rater reliability of rigidity assessment and have found it to be anywhere from “excellent” (Rabey et al., 1997), to “moderate” (Richards et al., 1994) and in between these extremes (Martínez-Martín, 1993; Prochazka et al., 1997). There is currently no standardized objective method to quantify rigidity but such a measure is fundamental for the assessment of response to therapies, especially since l-dopa affects rigidity more than other signs such as tremor (Langston et al., 1992; Fung et al., 2000). Several groups of researchers and clinicians have developed quantifiable rigidity measurements such as using a rigidity quantification device (Prochazka et al., 1997), assessing the mechanical resonant frequency (Lakie et al., 1984) and impedance (Patrick et al., 2001), and evaluating joint surface EMG and kinetic recordings (Endo et al., 2009). However, most of these techniques require a complex mechanical apparatus and/or measurement devices limiting clinical use. While more extensive work will be required to definitively establish if damping ratio provides an easily implemented rigidity assessment, the advantage of such a measure is that it is related to actual motor performance and hence more relevant to overall disability.

There are a number of limitations to our study. Our functional imaging data were gathered while subjects were actively engaged in performing a behavioral task while the rigidity scores were assessed during the passive movement of the limbs. However, as we previously noted, motor activation is often performed to augment rigidity, so we do not believe that this altered our interpretations. Rigidity is primarily a motor sign related to the integrity of the motor system, therefore altered brain activity may be most evident during actual motor performance. Nevertheless, future studies may employ a larger sample size and focus on resting state functional MRI to determine what resting connectivity patterns are related to rigidity.

## Conclusion

Results from our study suggest that rigidity is associated with widespread changes in the brain, as opposed to a single discrete locus. In addition, our results suggest that damping ratio may be an objective surrogate of the important clinical sign of rigidity.

## Conflict of Interest Statement

The authors declare that the research was conducted in the absence of any commercial or financial relationships that could be construed as a potential conflict of interest.
